# Simultaneous rib osteosynthesis and thoracoscopic suturing of diaphragm at patient with severe chest trauma and respiratory insufficiency

**DOI:** 10.1186/1749-8090-8-S1-P37

**Published:** 2013-09-11

**Authors:** A Benyan, E Korymasov, S Pushkin

**Affiliations:** 1 Samara Regional Clinical Hospital, Samara, Russian Federation; 2 Samara State Medical University, Samara, Russian Federation

## Background

Severity of respiratory insufficiency at severe chest trauma is determined by many pathogenic mechanisms, including multiple rib fractures, ARDS, rupture of diaphragm. In such cases only internal pneumatic stabilization didn`t solve that problem.

## Methods

We present a case report of 42-years old man, who had sustained an injury result of pedestrian accident. The investigation revealed multiple fractures of 9 left ribs forming flail chest, rupture of left cupula of diaphragm with hemopneumothorax, left lung contusion and fracture of left clavicle. All this caused severe respiratory insufficiency.

## Results

At first pulmonary ventilation was started and thoracocentesis with draining were performed. Then 4 rib osteosynthesis using Matrix Rib, thoracoscopy with sanation of hemothorax and suturing of diaphragm, and osteosynthesis of clavicle have been done. After operation patient was 36 hours on assisted ventilation. There were no complications in postoperative period. Patient was discharged from hospital in satisfactory condition on 14th day after operation.

## Conclusions

Synchronous repair of injured organs of chest is indicated procedure for it prevents many severe complications including respiratory insufficiency.

